# In Vivo Efficacy of Amphotericin B against Four *Candida auris* Clades

**DOI:** 10.3390/jof8050499

**Published:** 2022-05-11

**Authors:** Lajos Forgács, Andrew M. Borman, Renátó Kovács, Dávid Balázsi, Zoltán Tóth, Bence Balázs, Chiu Chun-Ju, Gábor Kardos, Ilona Kovacs, László Majoros

**Affiliations:** 1 Department of Medical Microbiology, Faculty of Medicine, University of Debrecen, Nagyerdei krt. 98., 4032 Debrecen, Hungary; forgacs.lajos.89@gmail.com (L.F.); kovacs.renato@med.unideb.hu (R.K.); balazsd95@gmail.com (D.B.); toth.zoltan@med.unideb.hu (Z.T.); balazs.bence@med.unideb.hu (B.B.); junchiou02@gmail.com (C.C.-J.); 2 Doctoral School of Pharmaceutical Sciences, University of Debrecen, Nagyerdei krt. 98., 4032 Debrecen, Hungary; 3 UK National Mycology Reference Laboratory, UK Health Security Agency, Science Quarter, Southmead Hospital, Bristol BS10 5NB, UK; andy.borman@nbt.nhs.uk; 4 Medical Research Council Centre for Medical Mycology (MRC CMM), University of Exeter, Exeter EX4 4QD, UK; 5 Faculty of Pharmacy, University of Debrecen, Nagyerdei krt. 98., 4032 Debrecen, Hungary; 6 Department of Metagenomics, University of Debrecen, Nagyerdei krt. 98., 4032 Debrecen, Hungary; kg@med.unideb.hu; 7 Department of Pathology, Kenézy Gyula Hospital, University of Debrecen, Nagyerdei krt. 98., 4032 Debrecen, Hungary; dr.kovacs.ilona@kenezy.unideb.hu

**Keywords:** *Candida auris*, amphotericin B, mouse, histopathology, in vivo

## Abstract

*Candida auris* is a multidrug-resistant fungus against which in some clinical situations amphotericin B (AMB) remains the alternative or first line drug. We compared daily 1 mg/kg of AMB efficacy in a neutropenic murine bloodstream infection model against 10 isolates representing four *C. auris* clades (South Asian *n* = 2; East Asian *n* = 2; South African *n* = 2; South American *n* = 4; two of which were of environmental origin). Five days of AMB treatment significantly increased the survival rates in mice infected with isolates of the East Asian clade, and 1 isolate each from the South African and South American clades (originated from bloodstream), but not in mice infected with the South Asian and 2 environmental isolates from the South American clades. AMB treatment decreased the fungal burden in mice infected with the 2 isolates each from East Asian and South African, and 1 out of 2 bloodstream isolates from South American clades in the hearts (*p* < 0.01), kidneys (*p* < 0.01) and brain (*p* < 0.05). AMB treatment, regardless of clades, significantly decreased colony forming units in the urine at day 3. However, histopathological examination in AMB-treated mice revealed large aggregates of yeast cells in the kidneys and hearts, and focal lesions in the cerebra and cerebelli, regardless of precise *C. auris* clade. Our clade-specific data confirm that the efficacy of AMB against *C. auris* is weak, explaining the therapeutic failures in clinical situations. Our results draw attention to the necessity to maximize the killing at the start of treatment to avoid later complications in the heart and central nervous system.

## 1. Introduction

*Candida auris* is an emerging multidrug-resistant fungus causing invasive infections and outbreaks worldwide [1,2]. Based on the whole genome sequencing, *C. auris* isolates were separated into five phylogenetically discrete clades (South Asian, East Asian, South African, South American and Iranian) with significant differences in their virulence, phenotypic characteristics and antifungal susceptibilities [3,4,5,6,7,8,9,10,11]. *C. auris* poses a therapeutic challenge not only due to its multidrug resistance but also because it causes infections in critically ill patients, especially those with underlying comorbidities (i.e., patients with diabetes mellitus, cardiovascular diseases, immunosuppressions, hematological malignancies and coronavirus disease 2019) [2,7,8,9,10,11]. Data from the Centers for Disease Control and Prevention revealed that 90%, 30% and <0.5% of *C. auris* isolates were resistant to fluconazole, amphotericin B and echinocandins, respectively, in the USA [2].

In our previous studies, we compared the virulence and antifungal susceptibilities of *C. auris* isolates belonging to the four main clades (South Asian, East Asian, South African and South American) [12,13,14]. Although the virulence of *C. auris*, regardless of clade, proved to be less than *C. albicans* in a neutropenic murine model, the highest virulence was found with the South American and South Asian clades both in lethality and fungal tissue burden experiments [12]. Anidulafungin, caspofungin, micafungin and rezafungin were fungistatic in killing studies either in RPMI-1640 or 50% serum against the four clades [13]. In contrast, amphotericin B (AMB) proved to be fungicidal at a clinically attainable concentration (1 mg/L) against 33%, 50%, 50% and 16.7% of isolates from the South Asian, East Asian, South African and South American lineages, respectively [14,15]. In contrast, Dudiuk et al. have found concentration-dependent but isolate-independent killing activity of AMB against nine Colombian isolates at >2 mg/L in their killing studies [16]. Moreover, in killing studies, we previously reported that the South Asian and South American clades, which were the most virulent in our neutropenic murine model, proved to be more resistant to AMB [12,14].

The aim of the current study was to determine the in vivo efficacy of AMB against *C. auris* isolates derived from the main four lineages (South Asian, East Asian, South African and South American) in a severely neutropenic murine model using lethality and fungal tissue burden approaches.

## 2. Materials and Methods

### 2.1. Isolates

Isolates of the four principal clades used (South Asian *n* = 2, East Asian *n* = 2, South African *n* = 2, South American *n* = 4) are derived from our previous study and are listed in Table 1 [14]. Two isolates from the South American clade were derived from hospital environments. *C. auris* isolates were identified by a combination of ribosomal DNA gene sequencing targeting the 28S rRNA and/or ITS1 regions, which was also used for clade delineation [5,6]. Two days before the experiments, isolates were sub-cultured using Sabouraud agar and screened on CHROMagar *Candida* (Becton Dickinson) to ensure purity of *Candida* isolates. AMB MICs and killing activity data were published earlier; MIC values were previously determined using the broth microdilution method according to CLSI M27-Ed4 document in RPMI-1640 and were lower than the tentative susceptibility breakpoint (1 mg/L) suggested by the CDC (Table 1) [2,14,17].

#### Mice and Immunosuppression

BALB/c male mice (23–25 g) were given cyclophosphamide (Endoxan, Baxter Hungary) 4 days before infection (150 mg/kg) and 1 day before infection (100 mg/kg), and also 2 and 5 days post-infection (100 mg/kg) in the fungal tissue burden experiments; in the lethality experiments, immunosuppression was continued by administration of 100 mg/kg cyclophosphamide every third day until the end of the experiment on the 21st day [12,17]. The Guidelines for the Care and Use of Laboratory Animals were strictly followed during maintenance of the animals; experiments were approved by the Animal Care Committee of the University of Debrecen (permission no. 12/2014).

### 2.2. Lethality Experiments

Mice (groups of ten mice/isolate) were infected intravenously through the lateral tail vein (day 0). A total of twenty groups of mice (AMB-treated and their untreated controls) were infected with 2 isolates each of the South Asian, East Asian and South African clades, and 4 isolates from the South American clade (2 of which originated from the hospital environment and 2 of which were from blood in patients with candidemia). The infectious dose was 10^7^ colony forming unit per mouse (CFU/mouse), administered in volumes of 0.2 mL. Inoculum densities were confirmed by plating serial dilutions on Sabouraud agar plates [12,17].

Treatment with amphotericin B (Fungizone^®^, Bristol-Myers Squibb, Uxbridge, England commercial preparation) began 24 h post-infection (day 1). Fungizone^®^ was purchased from our University Pharmacy and reconstituted according to the recommendation of the manufacturer. Daily 1 mg/kg of AMB was given intraperitoneally in a 0.5 mL volume for five days [18,19]. As higher daily doses of AMB (i.e., 2 and 5 mg/kg) immediately produced severe visible side effects (tremor, bulging eyes) in mice, only 1 mg/kg dose was used. Control groups were given saline.

Mice were monitored at least twice a day for lethality for 21 days. Animals that became immobile or showed signs of severe illness were terminated and recorded as dying on the following day. Dead animals (2–4 mice from each clade) including AMB-treated mice from the East Asian and South African clades with ataxia at 17–21 days were dissected for histopathology. Survival rates were compared using the Kaplan–Meier logrank test. Statistical tests were performed in Graph Pad 6.0.3 [19].

### 2.3. Fungal Tissue Burden Experiments

The experimental design was similar to that employed in lethality experiments. The infectious dose was decreased to 8 × 10^6^ CFU/mouse in each clade to avoid very early death. Each group included 12 mice. On day 6, six mice were sacrificed; both kidneys, the heart and the brain were removed from each animal, weighed and homogenized aseptically in 1 mL of saline; the resulting tissue suspension was serially diluted. Fungal tissue burden was determined by quantitative culturing. The lower limit of detection was 100 CFU/g of tissue. Mean fungal tissue burdens produced by the same organs were compared using the Kruskal–Wallis test with Dunn’s post-test [12,19].

### 2.4. Urine Collection

During intraperitoneal inoculation, personal protective equipment (i.e., gloves, clothes) were always urinated on by mice, which provided opportunity to collect urine samples of the mice for culturing. Urine was obtained on day 2 in the lethality experiments with isolates 196 (South Asian clade), 12,372 (East Asian clade), 204 (South African clade), I-156 (bloodstream isolate from the South American clade) and 13,112 (environmental isolate from the South American clade); 6 mice were sampled in each AMB-treated and each control group. Because of the poor condition of mice 4–5 days post-infection, urine was collected only on day 2. After disinfection of the periurethral region, the fresh urine samples were obtained by gentle transabdominal pressure over the bladder and were collected in a disposable, sterile plastic Petri plate. One micturation produced 50–80 µL of urine which was immediately serially diluted and inoculated onto Sabouraud agar for quantitative culture. After 2 days of incubation, CFU/mL were calculated, and AMB-treated and control groups were compared using the Student’s *t*-test.

### 2.5. Histopathology

Two to three mice from each AMB-treated group derived from the fungal tissue burden experiments were used for histopathological examination on day 2 and 6. Nineteen AMB-treated and nine control mice freshly found dead in the lethality experiments were dissected and analyzed similarly. Organs (heart, both kidneys and brain) were fixed in formalin and embedded in paraffin. Tissue sections (4 µm) were stained with hematoxylin-eosin and Periodic Acid Schiff (PAS). Hearts were also stained with Mallory’s phosphotungstic acid hematoxylin (PTAH) [12].

## 3. Results

### 3.1. Lethality Experiments

#### 3.1.1. South Asian Clade

**Figure 1:**
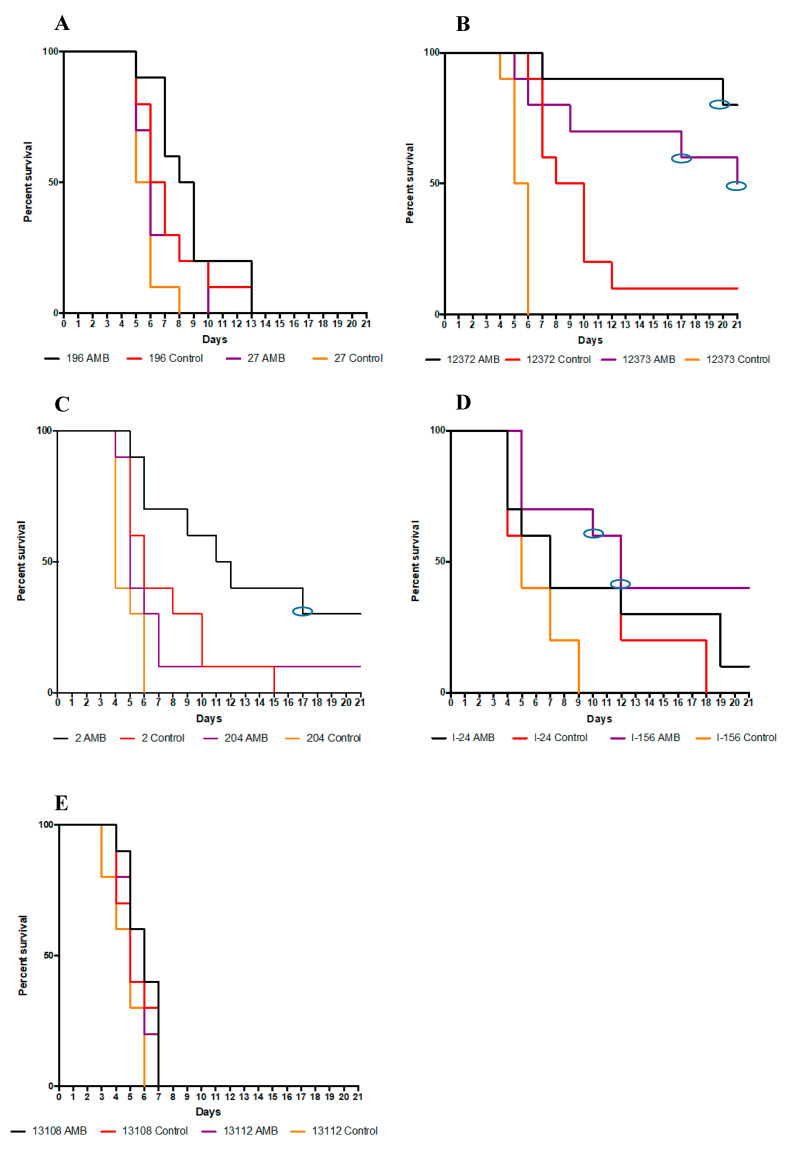
Survival of neutropenic AMB-treated and control BALB/c mice infected with the isolates from the four *Candida auris* clades: isolates 27 and 196 (**A**) (South Asian clade), isolates CBS 12,372 {12,372} and CBS 12,373 {12,373} (**B**) (East Asian clade), isolates 2 and 204 (**C**) (South African clade) and isolates I-24 and I-156 (**D**) (South American (clade). Two environmental isolates, CDC B-13,108 {13,108} and CDC B-13,112 {13,112} (**E**), from the South American clade were also tested. The infectious dose was 10^7^ CFU/mouse. Daily 1 mg/kg of amphotericin B (AMB) treatment began 24 h postinfection. After 21 days the survival rate was analyzed by Kaplan–Meier test. Blue ellipses in survival curves in cases of mice infected with isolates 12,372 and 12,373 (East Asian clade) (**B**), isolate 2 (South African clade) (**C**) and isolate I-156 (South American clade) (**E**) indicate that these mice showed ataxia 2–3 days before their death.

AMB treatment did not increase the survival rate in mice infected with either of the *C. auris* isolates of the South Asian clade (*p* values in cases of isolates 196 and 27 were 0.2004 and 0.1459, respectively) (Figure 1A). The lethality rates in AMB-treated mice infected with isolates 196 and 27 were 40% and 70%, respectively, at day 7 which increased to 100% by day 13.

#### 3.1.2. East Asian Clade

Daily AMB treatment significantly increased the survival rates in mice infected with isolates 12,372 (*p* = 0.0009) and 12,373 (*p* = 0.0005); at the end of the experiment (day 21) the survival rates were 80% and 50%, respectively (Figure 1B). One (which died on day 20) and two (which died on days 17 and 21) AMB-treated mice infected with isolates 12,372 and 12,373, respectively, showed ataxia 2–3 days before their death. Moreover, 3 out of 8 and 2 out of 5 surviving mice infected with isolates 12,372 and 12,373, respectively, also showed ataxia (Figure 1B). Control dead mice never showed signs of central nervous system involvement (CNS) before their death.

#### 3.1.3. South African Clade

AMB treatment significantly increased the survival of mice infected with isolate 2 (*p* = 0.0189), but not in the case of isolate 204 (*p* = 0.0595) (Figure 1C). The survival rates in AMB-treated mice with isolates 2 and 204 were 70% and 10%, respectively, at day 7. The AMB-treated mouse infected with isolate 2 that died on day 17 showed ataxia 3 days before its death (Figure 1C).

#### 3.1.4. South American Clade

AMB treatment in mice infected with bloodstream isolates from Israel increased the survival only in case of isolate I-156 (*p* = 0.0017) (Figure 1D). Two AMB-treated mice infected with isolate I-156 showed ataxia before their death (Figure 1D, days 10 and 12).

In mice infected with environmental isolates, AMB treatment could not prevent the rapid death observed among control as well as in treated mice (*p* values were 0.4469 and 0.1988 for isolates 13,108 and 13,112, respectively); all control and AMB-treated mice died by day 7 (Figure 1E).

### 3.2. Fungal Burden Experiments

All infected mice survived until the end of experiments (day 6).

#### 3.2.1. South Asian Clade

**Figure 2:**
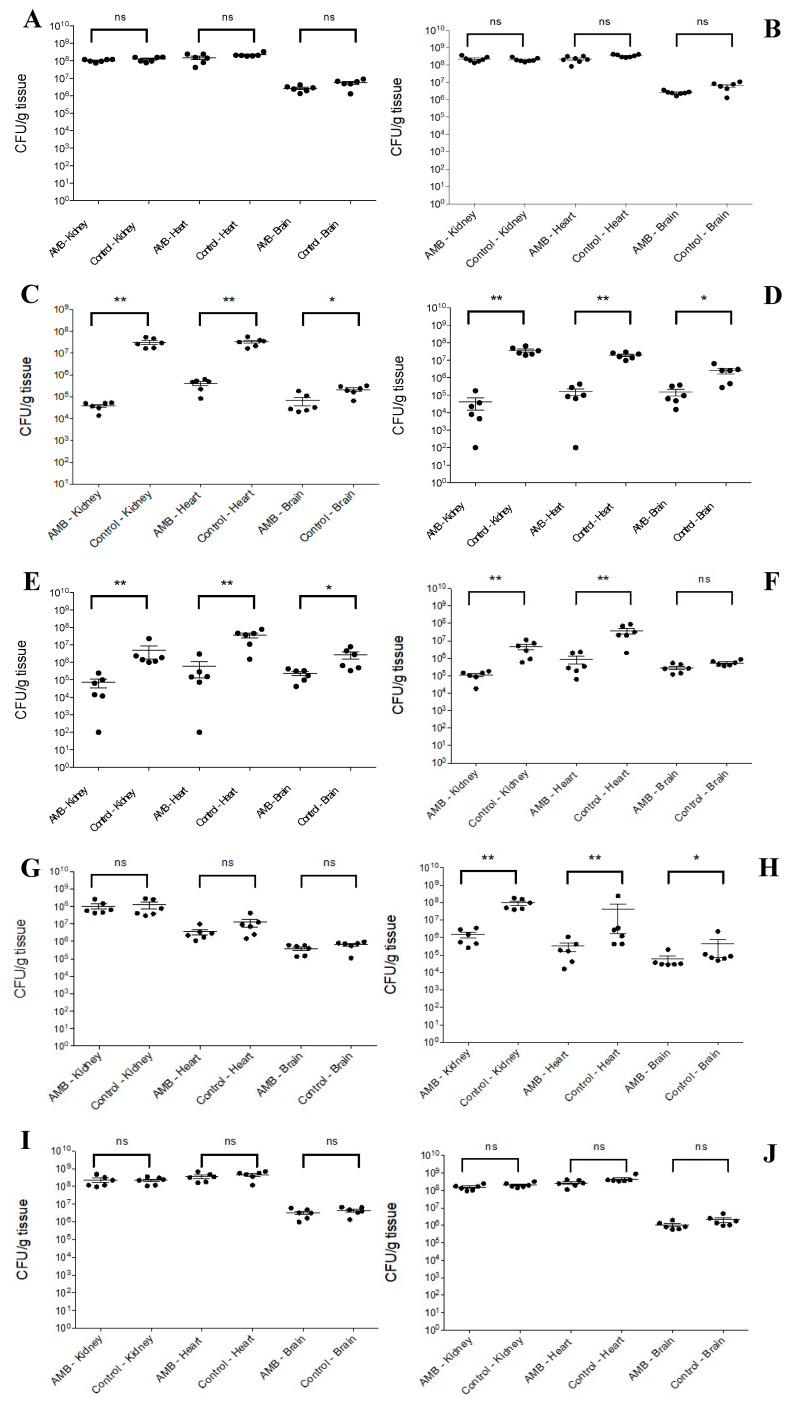
Fungal kidneys, heart and brain burdens were determined with 2-2 clinical isolates from the South Asian {isolates 27 (**A**) and 196 (**B**)}, East Asian {isolates CBS 12,372 (**C**) and CBS 12,373 (**D**)}, South African {isolates 2 (**E**) and 204 (**F**)} and South American {isolates I-24 (**G**) and I-156 (**H**)} clades at day 6. Two environmental isolates CDC B-13108 (**I**) and CDC B-13112 (**J**) from the South American clade were also tested. The infectious dose was 8 × 10^6^ CFU/mouse. Daily 1 mg/kg of amphotericin B (AMB) treatment began 24 h post-infection. The bars represent the medians. Level of statistical significance is indicated at *p* < 0.05 (*), *p* < 0.01 (**). NS corresponds to non-significant.

AMB treatment, regardless of isolate, did not cause statistically significant fungal burden decreases in the examined organs (*p* > 0.05, for all organs) (Figure 2A,B); the mean fungal burdens were higher than 10^8^, 10^8^ and 10^6^ CFU/g in the kidneys, hearts and brains, respectively.

#### 3.2.2. East Asian Clade

AMB produced more than 2 logs CFU/g decreases in fungal burden in the kidneys (*p* < 0.01) and hearts (*p* < 0.01) of mice infected with isolates 12,372 (Figure 2C) and 12,373 (Figure 2D). AMB generated statistically significant CFU decreases in the brains (*p* < 0.05 with both treated groups), but the mean fungal burdens in the brains were higher or close to 10^5^ CFU/g. In the case of isolate 12,373 (Figure 2D) in one mouse each, the kidneys and the heart were sterile (CFU number was lower than 100 CFU/g). The lowest mean fungal burden was observed in the kidneys (<10^5^ CFU/g).

#### 3.2.3. South African Clade

AMB treatment (with the exception of the brain in mice infected with isolate 204) produced statistically significant CFU decreases in the organs with the two isolates (Figure 2E,F). The kidneys and the heart were sterile in one mouse each in mice infected with isolate 2 (Figure 2E). The mean fungal brain burdens were >10^5^ CFU/g in AMB-treated mice with both isolates.

#### 3.2.4. South American Clade

In mice infected with isolate I-24, CFU decreases were never observed in any of the examined organs (*p* > 0.05) (Figure 2G). In contrast, in case of mice infected with isolate I-156, AMB decreased the fungal burdens in the kidneys (*p* < 0.01), heart (*p* < 0.01) and brain (*p* < 0.05); CFU decreases were near 2 logs in the kidneys and heart (Figure 2H).

In mice infected with environmental isolates of the South American clade, the fungal burdens in the kidneys, hearts and brains in AMB-treated mice were not reduced when compared with their control untreated animals (*p* > 0.05) (Figure 2I,J).

### 3.3. Urine Culture

**Figure 3:**
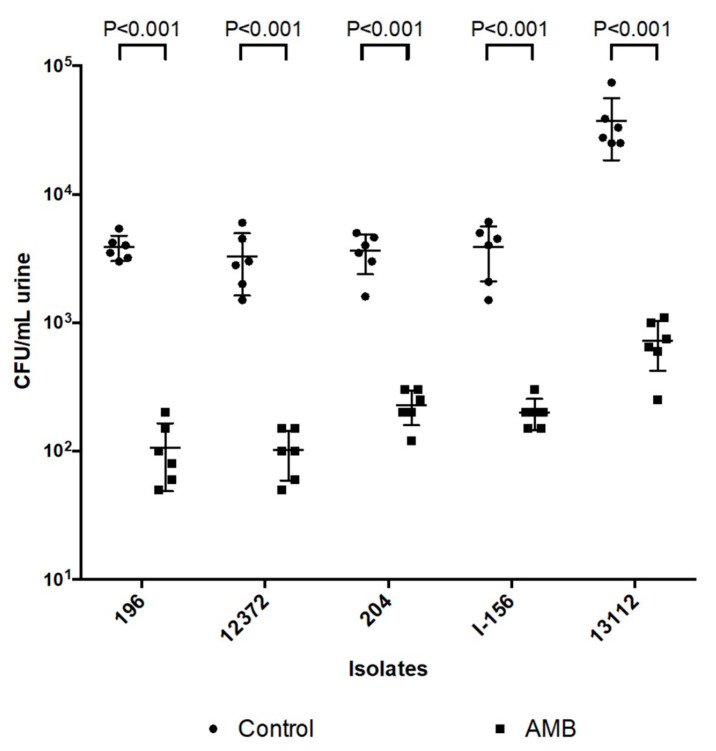
The effect of 2 days amphotericin B (AMB) treatment on the CFU in the urine in mice infected with isolates 196 (South Asian clade), 12,372 (East Asian clade), 204 (South African clade), I-156 (bloodstream isolate from the South American clade) and 13,112 (environmental isolate from the South American clade). Urine was collected on day 3. The bars represent the medians. Level of statistical significance is indicated at *p* < 0.001.

Urine of control mice infected with isolates 196 (South Asian clade), 12,372 (East Asian clade), 204 (South African clade), I-156 (bloodstream isolate from the South American clade) and 13,112 (environmental isolate from the South American clade) contained 3.2–5.4 × 10^3^, 2–6 × 10^3^, 1.6–4 × 10^3^, 1.08–6.1 × 10^3^ and 8.8 × 10^3^–7.4 × 10^4^ CFU/mL, respectively, of *C. auris*. Two days AMB treatment (day 3) produced at least 1 log mean CFU decreases in mice compared to controls (*p* < 0.001 for all cases) (Figure 3).

### 3.4. Histopathology

#### 3.4.1. Lethality Experiments

##### South Asian Clade

**Figure 4:**
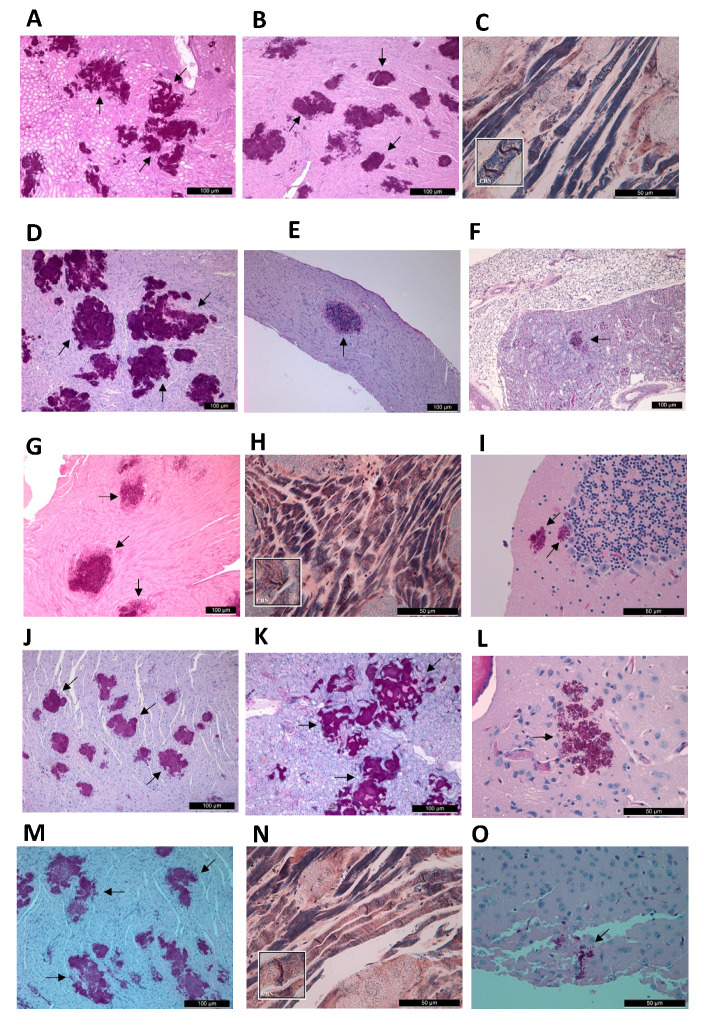
Histopathological examination of the heart, kidney and brain with Periodic Acid Schiff or Mallory’s PTAH staining from amphotericin B (AMB) treated mice found dead after intravenously challenged with *Candida auris* isolates. In mice infected with isolate 196 (South Asian clade) which died on day 5, kidneys (**A**) showed large multifocal fungal lesions, fungal cells were detected in the parenchyma, necrosis occurred in the tubular tissue, but glomeruli were not invaded. In the heart (**B**), *C. auris* produced large aggregates and coagulative necrosis of myocytes. Mallory’s PTAH staining always revealed contraction band necrosis (myofibrillar degeneration) (**C**). In the case of mice infected with isolate 12,372 (East Asian clade) which died on day 6, in the hearts multifocal (**D**) or only focal (**E**) fungal infiltrates were found. In the kidneys (**F**) small lesions were detectable (black arrow). In mice infected with isolate 204 (South African clade) which died on day 5, 4–5 aggregates were seen in the hearts (**G**) with contraction band necrosis (**H**). In the cerebellum (**I**), one large and one smaller PAS positive fungal lesions were seen. In mice infected with isolate I-156 (South American clade) which died on day 5, multifocal, large aggregates were seen in the hearts (**J**) and kidneys (**K**) with abundant yeast cells in the cerebrum (**L**). In mice infected with isolate CDC B-13108 which died on day 4, the heart showed large and small multifocal infiltrates (**M**) with contraction band necrosis (**N**). In the cerebrum (**O**), yeast cells were visible. Fungal lesions were indicated with black arrows. Magnification, (**A**,**B**,**D**–**G**,**J**,**K,M**) ×100, (**C**,**H**,**I**,**L**,**N,O**) ×400.

The dead AMB-treated mice (days 5–6) showed large multifocal fungal infiltrates in their kidneys (Figure 4A) and hearts (Figure 4B). Contraction band necrosis was observed in all hearts with Mallory’s PTAH staining (Figure 4C). CNS was always invaded. The hearts (Figure S1A) and kidneys (Figure S1B) of the controls (days 5–6) were similar to the AMB-treated dead mice, and involvement of the cerebra and/or cerebelli was also found in all control mice.

##### East Asian Clade

**Figure 5:**
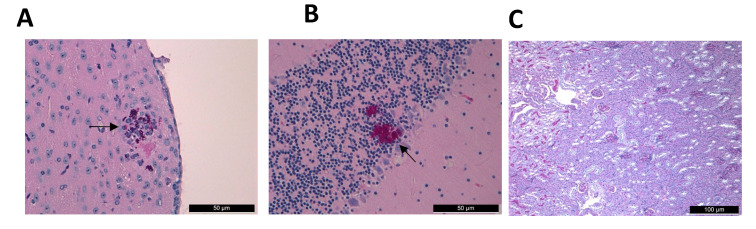
Histopathological examination of the cerebrum, cerebellum and kidney with Periodic Acid Schiff staining from a neutropenic, amphotericin B treated, mice infected with isolate 12,372 (East Asian clade) which died on day 20. The cerebrum (**A**) and cerebellum (**B**) showed yeast cells, but kidneys (**C**) were sterile, with normal histology without fungal cells. Fungal lesions were indicated with black arrows. Magnification, ×100.

Histopathological examination in AMB-treated mice in the first 9 days of infection showed large multifocal (Figure 4D) or focal (Figure 4E) fungal infiltrates in the hearts and kidneys (Figure 4F) as well as the invasion of the CNS. Untreated controls always exhibited large multifocal fungal infiltrates in the hearts (Figure S1C) and kidneys (Figure S1D). CNS involvement was similar to that seen is treated mice. AMB-treated mice that died on day 17 and day 21 (in case of isolate 12,373), and day 20 (in case of isolate 12,372) showed cerebra (Figure 5A) and cerebella (Figure 5B) involvement, but fungal cells were not apparent in their kidneys (Figure 5C) and hearts.

##### South African Clade

In the hearts (Figure 4G) and kidneys, numerous aggregates were observed in AMB-treated mice that died on days 4–7. However, the number of aggregates was lower compared to the South Asian clade (Figure 4A,B). Contraction band necrosis was always observed in the hearts (Figure 4H). In the cerebra and cerebella (Figure 4I) fungal cells were observed. The histopathology of the hearts (Figure S1E), kidneys (Figure S1F) and central nervous system in control mice were similar to AMB-treated mice. In the case of a mouse infected with isolate 2 that died on day 17, the cerebrum and cerebellum showed yeast cells, but the heart and kidneys showed normal histology without fungal cells.

##### South American Clade

AMB-treated mice infected with the 2 bloodstream isolates which died on days 4–5 showed multifocal fungal infiltrates in the hearts (Figure 4J), kidneys (Figure 4K) and invasion of the cerebra (Figure 4L) and cerebella. Contraction band necrosis was observed in dead mice. The histopathological findings in cases of the environmental isolates in the hearts (Figure 4M,N), kidneys and CNS (Figure 4O) were similar on day 4. In control mice, the lesions in the hearts (Figure S1G) and kidneys (Figure S1H) were larger, and fungal cells were found in mice infected with isolate 13,108 in the cerebrum on day 3.

#### 3.4.2. Fungal Burden Experiments

**Figure 6:**
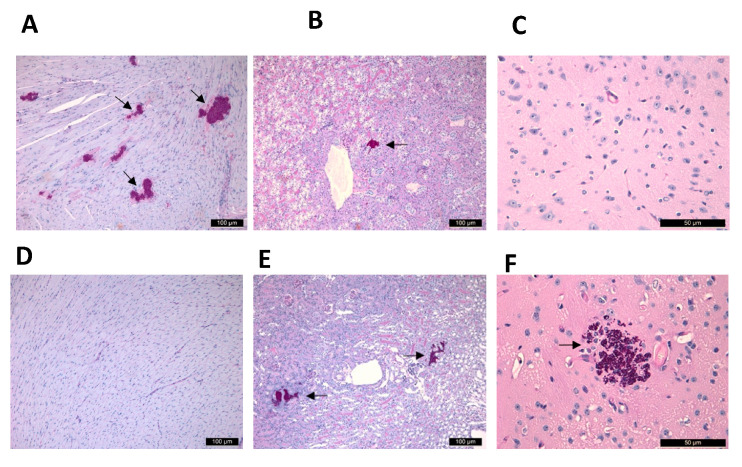
Histopathological findings of the heart, kidney and cerebrum with Periodic Acid Schiff staining from fungal burden experiments. Neutropenic mice infected with isolate I-156 (South American clade) sacrificed and dissected on day 2 showed small fungal foci in the hearts (**A**) and kidneys (**B**), but the cerebrum (**C**) did not contain fungal cells. The histopathology regardless that mice were AMB-treated or not was the same for all isolates and clades on day 2. In case of a mouse infected with isolate 12,372 (East Asian clade) and treated with 1 mg/kg of AMB, on day 6 the heart showed normal histology without fungal involvement (**D**), but the kidney (**E**) and cerebrum (**F**) showed fungal cells. Fungal lesions were indicated with black arrows. Magnification, ×100.

All examined AMB-treated and control mice infected with the isolates from the four clades showed similar histopathology on day 2: multifocal, relatively small fungal infiltrates in the hearts (Figure 6A) and kidneys (Figure 6B) without CNS involvement (Figure 6C). In contrast, cerebra and cerebella showed yeast cells in AMB-treated mice infected with any isolates on day 6. Two out of three mice infected with isolate 12,372 (East Asian clade) showed yeast cells only in their cerebra and cerebella but not in the kidneys or heart. In the case of the third mouse, the kidneys (Figure 6E) and cerebrum (Figure 6F) showed fungal involvement, but the heart did not (Figure 6D). In one out of three mice infected with the South African clade, cerebral involvement was detected but not kidney or heart involvement.

## 4. Discussion

The increase in non-albicans species among *Candida* infections in recent years is worrying due to their higher antifungal resistance [20,21,22]. In this study daily 1 mg/kg of AMB showed clade- and isolate-dependent activity against *C. auris* in our severely neutropenic murine model. The intravenous infection rapidly spread into the internal organs including the CNS; AMB treatment increased mouse survival rates significantly only in the cases of the 2 isolates from the East Asian, 1 from the South African and 1 from the South American clade (Figure 1B–D). Survival data were in agreement with fungal burden data. Five days of AMB treatment in cases of the otherwise less virulent East Asian and South African clades were able to decrease the fungal tissue burdens in the hearts and kidneys, as supported by histopathological findings, leading to prolonged survival of mice. AMB was ineffective in sterilization of the CNS as revealed by the high (at least 10^5^ CFU/g) fungal burden at day 6 and ataxia regardless of clade among mice in the lethality experiments. Notably, in cases of the most virulent South Asian and South American clades, clinical signs of CNS involvement (i.e., torticollis, ataxia) were rarely or never noticed at all among AMB-treated mice, as the severe heart muscle damage led to early death, before signs of CNS involvement could develop. Alarmingly, mice infected with the environmental isolates proved to respond poorly to treatment with AMB.

Our results are consistent with the findings of other authors. Lepak et al. found that 1.25, 5 and 20 mg/kg of AMB were fungistatic in fungal kidney burden experiments against 8 out of 9 *C. auris* isolates (representing the four main clades) in a neutropenic murine model 2 h post-infection [23]. AMB showed the best effect (more than 1 log CFU decreases) against a single Japanese isolate (East Asian clade). Ghannoum and colleagues determined the efficacy of AMB in the lethality and fungal kidney burden experiments against 1 isolate of *C. auris*. They found that the comparators (liposomal AMB, and micafungin and rezafungin) showed significantly better activity in a neutropenic murine model than conventional AMB 2 h post-infection [24,25]. However, they did not examine the efficacy of AMB in the treatment of fungal myocarditis and CNS infection caused by *C. auris*. The lack of similar studies with the efficacy of AMB against the four main *C. auris* clades precludes comparison to our results.

A limitation of this study is that the fungal burden was tested in the kidneys, the heart and the brain. Other authors found that intravenous *C. auris* infection produced high fungal burden in neutropenic mice in the lungs, cecum, uterus and stomach [26]. However, previous studies have shown that the heart and kidneys are the main targets in cases of invasive infections caused by *C. auris*, together with the CNS involvement [12,27,28,29]; the fungal burden in these sites, especially in the heart, is the primary cause for death in mice [12]. The strength of our study is that the in vivo efficacy of AMB was tested against the four main clades both in lethality and fungal burden experiments including the effect on the CFU in the urine after 2 days AMB treatment. Moreover, the frequently performed histopathological examinations provided important insights into the early phase of the pathogenesis of invasive *C. auris* infections. However, the fifth clade was not represented.

Candidemia is the most common manifestation of *C. auris* infection and requires immediate treatment with fungicidal antifungal agents (i.e., echinocandins or AMB) [1,3,7,8,9,10,11]. Treatment failures with echinocandins and AMB were observed in patients with persistent or recurrent candidemia as a direct consequence of the inadequate clearance of the fungus [30,31]. Metastatic dissemination may further complicate the incomplete eradication leading to endophthalmitis, spondylodiscitis and endo/myocarditis [30]. Although CNS involvement is rare, cases of ventriculitis have been observed among adult patients with neurosurgical interventions or subarachnoid hemorrhages [32,33]. Moreover, an Iranian infant developed meningitis due to a *C. auris* isolate belonging to the South Asian clade [34].

Our results strongly suggest that heart damage is primarily responsible for death among AMB-treated and control mice (Figure 4C,H,N). Histopathological findings on day 2 (Figure 6A–C) confirmed our previous observations that intravenous inoculation of *C. auris* cells produced high fungal tissue burdens in the heart and kidneys 2 days post-infection [12]. AMB treatment in cases of the more virulent South Asian and South American clades was unable to clear or decrease the fungal burden in the examined organs as revealed by the histopathological studies in dead mice on days 3–4 (contraction band necrosis in the hearts and massive fungal infiltration of the kidneys and brains) (Figure 4A–C,J–O). Though we did not measure AMB concentrations in the serum, hearts, kidneys and brains, which may be regarded as another limitation of the study, other authors have found 0.1 mg/L, 0.7 µg/g and 0.3 µg/g AMB concentrations in the serum, hearts and kidneys, respectively, in mice 4 h post-inoculation of 1 mg/kg of AMB [35]. Moreover, AMB is a highly protein-bound drug, further decreasing the free, thus biologically active AMB concentrations in the serum as well as in tissues, explaining the weak efficacy of AMB [15]. This is especially prominent against the environmental isolates from the South American clade, the least susceptible isolates in killing studies [14]. In contrast, AMB treatment significantly decreased the fungal kidney and heart tissue burdens in mice infected with less virulent isolates from the East Asian and South African lineages by day 6; mice could survive even after 21 days with or without clinical signs of meningitis. It is noteworthy that AMB-treated mice that died and were dissected at the end of the lethality experiments did not show fungal cells in their hearts and kidneys (Figure 5C), but their cerebra (5A) and/or cerebella (5B) always showed fungal cells. Our histopathological results suggest that at least 3 days are needed to invade the CNS by *C. auris*. In contrast to Singh et al., in this study the fungal cells were found in the brain tissues, not in the capillaries [28]. These clade-specific data suggest that the concentration of AMB was high enough in the first 6 days of the experiment to decrease or, sometimes, eradicate the fungal cells from the heart, kidneys and urine (Figure 2C–F,H and Figure 3), but not from the CNS (Figure 6F) [15,16,35,36].

*C. auris* strains isolated from the patients were genetically identical to strains from hospital surfaces and healthcare workers suggesting that source of *C. auris* may be healthcare workers or the hospital environment [7]. Recent data confirmed the importance of *C. auris* environmental isolates, as Arora et al. isolated *C. auris* from the sandy beach and a tidal swamp in India. These environmental strains were genetically close to the clinical isolates from India (South Asian clade) and, with the exception of one isolate, showed multidrug resistance [37]. Moreover, *C. auris* was isolated from the surfaces of stored apples; these strains were closely related to strains from patients, hospitals in India as well as to clinical strains from other parts of the world (South Asian clade) [38]. As hospital environmental isolates may be the source of colonization or infection in critically ill patients, antifungal susceptibility testing of these isolates is mandatory. Notably, daily 1 mg/kg AMB was totally ineffective against the two Colombian environmental isolates (South American clade) in our neutropenic murine model, suggesting that environmental strains may pose a therapeutic challenge.

The previously reported weak killing activity of AMB in vitro was confirmed in our in vivo model [14,16], suggesting that during or after *C. auris* candidemia, clinicians should regularly check the internal organs with medical imaging (i.e., ultrasound, computed tomography) (heart, kidneys, lungs, etc.) to detect early the signs of disseminated infection. Routine urine culture seems to be useful in all clinical situations when invasive *C. auris* infection is suspected as in this study urine was positive 3 days post-infection. However, urine culture positive for *C. auris* may be the direct consequence of contamination from the periurethral region. Thus, repeated blood and urine culture may help to differentiate between contamination and candiduria with or without bloodstream infection. Persistent or recurrent candidemia due to the weak killing activity of antifungals increases the risk for heart, eye or CNS infection leading to further complications [8,9,10,11,30,31,32,33,34,39]. As AMB is still the first therapeutic choice in certain clinical situations (i.e., neonates and infants < 2 months of age, meningitis, endophthalmitis and urinary tract infections), physicians should be aware of inadequate fungal killing with normal doses of AMB or even echinocandins [2]. Higher daily doses with echinocandins or lipid-associated AMB may prevent these complications during candidemia (i.e., meningitis, endophthalmitis and endo/myocarditis). Moreover, combinations of AMB with echinocandins frequently led to complete eradication of *C. auris*, even from the CNS [10,32,33,34]. However, AMB treatment is limited by infusion-related acute toxicity (i.e., chest pain, fever and hypoxia) and dose-dependent nephrotoxicity (i.e., renal insufficiency, azotemia and hypokalemia) [15].

## 5. Conclusions

In summary, our results show that *C. auris* can invade the hearts, kidneys and CNS despite early initiation of AMB treatment. Daily 1 mg/kg of AMB increased the survival rate and decreased the fungal tissues burden only in the cases of the less virulent and in vitro more AMB-susceptible East Asian and South African clades. However, against the other clades, the efficacy of AMB is questionable. In cases of suspected invasive *C. auris* infection, high daily doses of echinocandins or AMB treatment should be initiated as soon as possible to protect patients from the dire consequences of fungal myocarditis or meningitis.

## Figures and Tables

**Table 1:** Characteristics of *Candida auris* isolates used in the study.

Clade	Isolate Number	Body Site
South Asian	27 (NCPF 89891)	Pleural fluid
	196	Blood
East Asian	12372 (CBS 12372)	Blood
	12373 (CBS 12373)	Blood
South African	2 (NCPF 8977)	Cerebrospinal fluid
	204	Tracheostomy
South American	13108 (CDC B-13108)	Hospital environment
	13112 (CDC B-13112)	Hospital environment
	I-24	Blood
	I-156	Blood

## Data Availability

Not applicable.

## Supplementary Materials

The following supporting information can be downloaded at: https://www.mdpi.com/article/10.3390/jof8050499/s1, Figure S1: Histopathological examination of the heart and kidney with Periodic Acid Schiff staining from neutropenic control, dead mice intravenously challenged with *Candida auris* isolates. In mice infected with isolate 196 (South Asian clade) which died on day 5, the heart (**A**) and kidney (**B**) showed large multifocal fungal lesions. In case of mice infected with isolate 12,372 (East Asian clade) which died on day 6, in the heart multifocal (**C**) fungal infiltrates were detectable. In the kidneys (**D**), large, confluent large lesions were seen. In mice infected with isolate 204 (South African clade) which died on day 5, large aggregates were seen in the hearts (**E**) and smaller aggregates were visible in the kidney (**F**). In mice infected with isolate CDC B-13108 (South American clade) which died on day 4, multifocal, large aggregates were seen in the hearts (**G**), and large and smaller aggregates in the kidneys (**H**). Magnification: ×100.
