# Supplementary figures and images for: In Vivo Efficacy of Amphotericin B against Four Candida auris Clades
Source: J Fungi (Basel). 2022 May 11;8(5):499. doi: 10.3390/jof8050499 (PMC9144575; doi:10.3390/jof8050499)

**A**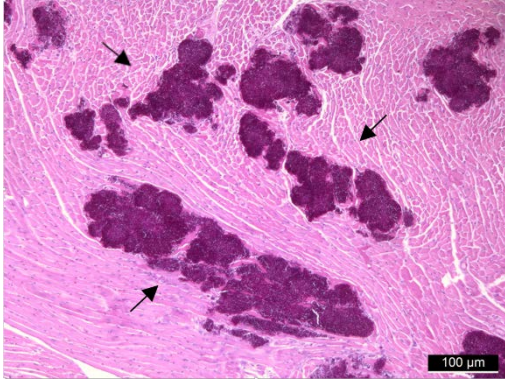**B**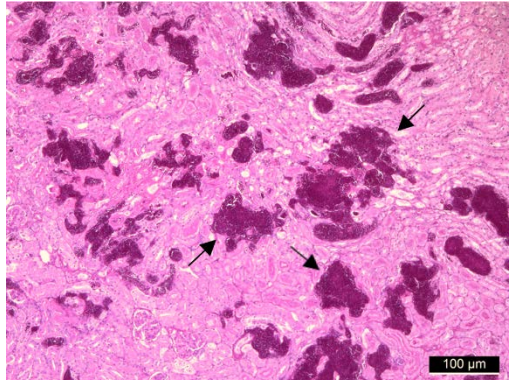**C**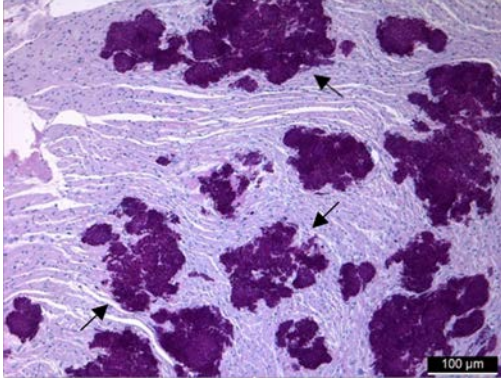**D**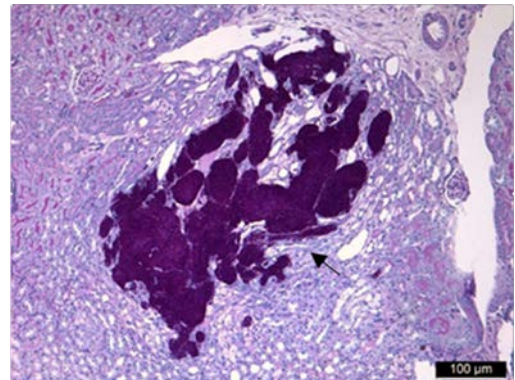**E**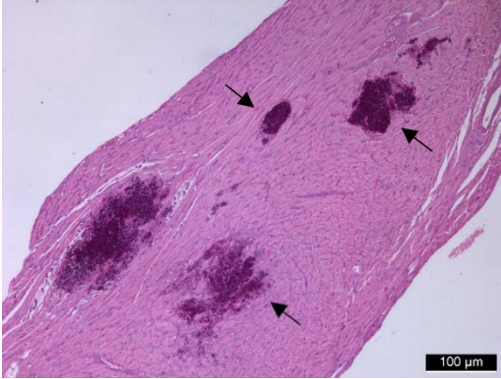**F**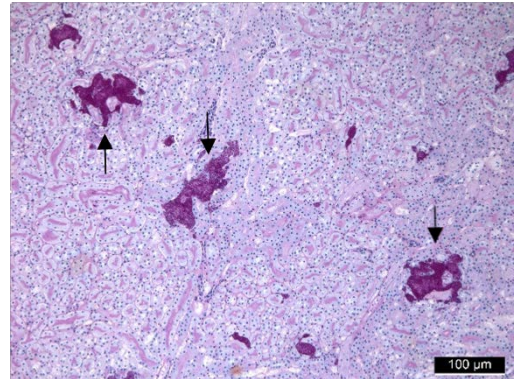**G**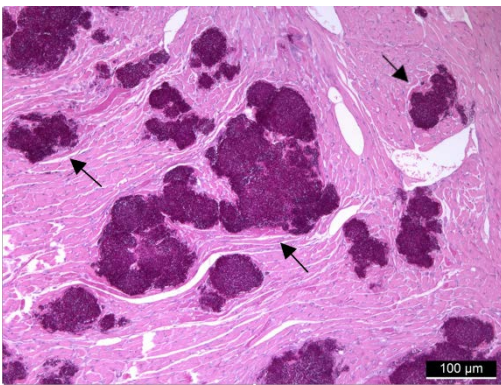**H**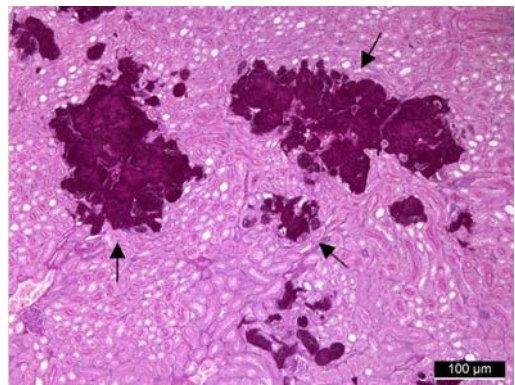

Supplement: Supplementary file 1 [file jof-08-00499-s001.zip › jof-1715094-SI.pdf]
